# Carcass and meat characteristics of Nellore young bulls fed diet using cottonseed cake as a replacer of the forage fiber source

**DOI:** 10.1038/s41598-024-58738-9

**Published:** 2024-04-05

**Authors:** Angelo Herbet Moreira Arcanjo, Luís Carlos Vinhas Ítavo, Camila Celeste Brandão Ferreira Ítavo, Marina Nadai de Bonin Gomes, Carlos Eduardo Domingues Nazário, Antonio Leandro Chaves Gurgel, Tairon Pannunzio Dias-Silva, Juliana Caroline Santos Santana, Manoel Gustavo Paranhos da Silva, Flávio de Aguiar Coelho, Estevão Lopes Miranda, Évelyn Silva de Melo Soares, Ana Hellen da Silva, Laura Beatriz Perez da Silva, Rafael Goes Cardoso Paro

**Affiliations:** 1 Faculty of Veterinary Medicine and Animal Science, Federal University of Mato Grosso do Sul, Campo Grande, MS Brazil; 2 West Unit, Getúlio Vargas Experimental Field, Agricultural Research Company of Minas Gerais, Uberaba, MG Brazil; 3 Institute of Chemistry, Federal University of Mato Grosso Do Sul, Campo Grande, MS Brazil; 4 Campus Professora Cinobelina Elvas, Federal University of Piaui, Bom Jesus, PI Brazil; 5 Faculty of Animal Science and Food Engineering, University of São Paulo, Pirassununga, SP Brazil

**Keywords:** Zoology, Animal behaviour, Animal physiology

## Abstract

The aim of this study was to assess the effects of substituting traditional forage fiber sources with cottonseed cake in the diet on both the quantitative and qualitative characteristics of carcass and meat in Nelore young bulls. Twenty-four Nelore steers starting with an average weight of 377.8 ± 43.5 kg, were individually housed in stalls and provided with individualized feeding over a 112-day confinement period. The study followed a completely randomized design with two treatments and 12 replications. The diets incorporated either whole plant corn silage (WPCS) and, cottonseed cake (CSC) as fiber sources, at a rate of 300 g/kg of dry matter. The CSC diet promoted higher carcass weight. Aging animal meat for seven days significantly decreased the shear force from 83.4 to 71.6 N. Although diets did not influence meat composition, WPCS diet provided higher concentrations of C16:1, C18:1n9c, C18:3n3, and C22:2 acid, and CSC diet higher concentrations of C15:0, C18:1n9t, C18:2n6c, and 20:3n3. The WPCS diet provided higher concentrations of monounsaturated fatty acids and ω9, and the CSC diet had higher concentrations of ω6 and ω6:ω3 ratio in meat. Cottonseed cake used as a fiber source increases the concentration of polyunsaturated fatty acids and ω6 fatty acids in the meat of young bulls finished in feedlot.

## Introduction

Over the past two decades, in Brazil, there has been a significant increase in the inclusion of concentrates in the feed of feedlot beef cattle, while there has been a corresponding decrease in the use of forages^1^. This change has led to the formulation of diets with a high starch content, providing greater energy availability^2^. Consequently, adjustments in cattle feeding management have been necessary to achieve higher production efficiency and meat quality^3,4^.

The greater energy input in the finishing phase of cattle, above the maintenance requirements, has the purpose of potentiating the fattening^3,5^. Considering that in this phase, following the plateau of the growth curve, there is a reduction in muscle tissue deposition and an increase in fat deposition ^6^. Thus, for the finishing phase of feedlot cattle, it is essential to provide more energy-dense diets, aiming to increase productivity indices and obtain better finished carcass, as well as meats with more marbling, characteristics valued by consumers^7^.

However, diets with high concentrate content, low fiber content, and particle size less than 8 mm can cause physically effective fiber deficiency (peNDF^8,9^), which alters rumination activities, chewing, and salivation^10,11^. In addition, ruminal environment acidification can lead to metabolic disorders such as ruminal acidosis and bloat, resulting in decreased microbial protein synthesis, particularly due to the decline in the population of fibrolytic bacteria^9,12^.

In this sense, there is a need to increase the energy density of feedlot cattle diets without reducing the planned levels of peNDF. Some by-products of the vegetable oil extraction industry have been used to improve the effectiveness of fiber in feedlot diets^13,14^. Cottonseed cake is a by-product present in ruminant feed^13–21^. Since it has a different nutritional profile from the preserved forage sources normally used in Brazilian feedlots, cottonseed cake can change the characteristics of the carcass and beef^22^. It is known that oilseeds added to the diet of animals increase the concentration of fatty acids beneficial to human health in sheep meat^23^, beef meat^22,24^ and bovine milk^25^. However, the effects of using cottonseed cake on meat quality are still scarce.

Additionally, the inclusion of cottonseed cake as peNDF in the diet of steers enhances feed efficiency, prolongs feeding time, and reduces rumination time and efficiency^21^. Therefore, cottonseed cake emerges as an alternative source of peNDF for feedlot beef cattle^21^. It is worth noting that substituting cottonseed cake for forage fiber sources brings logistical benefits to the feedlot, as it reduces the need for machinery for planting, harvesting, storage, and provision of forages. Another relevant aspect is that using agro-industrial by-products in animal feed enables the proper disposal of these residues, directly contributing to the sustainability of animal and agro-industrial production.

It should be noted that the oil extraction process may vary depending on the method and the oilseed. The physical method (cold pressing) results in a by-product rich in fatty acids. Ítavo et al.^26^, investigating the composition and quality of different oilseeds, observed that the cakes resulting from the pressing process have higher levels of total lipids, practically twice the content. It is important to emphasize that the incorporation of lipid-rich foods in the diet of ruminants can alter the ruminal microbiota^27,28^. This is because lipids have the potential to modify the permeability of the ruminal bacteria's plasma membrane, resulting in a reduction in microbial protein synthesis. Additionally, an excess of lipids can significantly decrease the ability of microorganisms to adhere to fiber particles. Thus, it is crucial to assess the appropriate levels of inclusion of cottonseed cake as a substitute for forage fiber sources.

Therefore, we hypothesized that adding cottonseed cake, which is rich in unsaturated fatty acids when included in forage-free diets for Nellore young bulls as a fiber source, could improve the quantitative and qualitative characteristics of the carcass and meat. Thus, the aim was to evaluate the quantitative and qualitative carcass and meat characteristics of Nellore young bulls confined and finished with a high-concentrate diet containing cottonseed cake or whole-plant corn silage as a fiber source.

## Results

The diet with whole plant corn silage (WPCS) promoted higher intake (*P* < 0.05) of dry matter (DM), organic matter (OM), crude protein (CP), neutral detergent fiber (NDF) and peNDF than the diet with cottonseed cake (CSC) (Table 1). Bulls fed the CSC diet had higher final body weight (FBW), hot carcass weight (HCW) and carcass yield (CY) and lower rib eye area (REA) (*P* < 0.05). There was no difference in the gain yield (GY) (*P* > 0.05) of bulls fed with the two fiber sources (Table 1).

**Table 1:** Quantitative and qualitative characteristics of carcasses of Nellore young bulls fed high-concentrate diet with different fiber sources.

	Fiber source		SEM	*P*-value
	WPCS	CSC		
Dry matter intake (kg/day)	10.5	8.8	0.60	0.0090
Organic matter intake (kg/day)	10.0	8.4	0.56	0.0088
Crude protein intake ((kg/day)	1.9	1.6	0.07	0.0341
Neutral detergent fiber intake (kg/day)	3.6	2.4	0.37	< 0.0001
Physically effective neutral detergent fiber (kg/day)	1.7	1.4	0.08	0.0418
Final body weight (kg)	525.8	535.7	31.49	< 0.0001
Hot carcass weight (kg)	288.4	299.5	18.65	< 0.0001
Carcass yield (%)	54.9	56.0	2.06	< 0.0001
Gain yield (%)	69.3	70.0	2.45	0.8534
Carcass Finishing (mm)	3.3	3.3	0.24	0.8961
Carcass Length (cm)	133.4	133.0	3.42	0.9043
Inner Depth (cm)	45.0	44.2	1.09	0.4619
External Depth (cm)	51.7	50.6	1.16	0.3489
Physiological Maturity (months)	21.5	19.7	3.25	0.5918
Conformation (score)	9.8	8.7	0.63	0.0938
Rib eye area (cm^2^)	76.5	68.6	2.73	0.0090
Marbling (score)	9.0	8.5	1.87	0.8110
Fat Distribution (score)	2.3	1.9	0.25	0.2037
Subcutaneous fat thickness (mm)	6.9	7.5	0.95	0.5402
pH	6.2	6.2	0.09	0.6951
Longissimus muscle color				
L*	39.7	38.1	2.33	0.5184
a*	21.3	21.0	1.57	0.8572
b*	11.0	10.7	0.77	0.7886
Fat color of Longissimus muscle				
L*	69.6	68.7	2.05	0.6556
a*	10.5	10.2	1.51	0.8585
b*	13.9	12.7	0.84	0.1524

*WPCS* diet with whole plant corn silage as fiber source, *CSC* diet with cottonseed cake as fiber source.

L* = luminosity; a* = red intensity; b* = yellow intensities.

There was no difference (*P* > 0.05) in the quantitative characteristics evaluated in the carcass typification (finish, length, internal and external depths, and conformation) of Nellore young bulls finished with different sources of fiber in the diet (Table 1).

The centesimal composition of the *Longissimus* muscle, including DM, mineral matter (MM), organic matter (OM), protein, and fat, showed no significant influence (*P* > 0.05) from the fiber source in the diets (Table 2).

**Table 2:** Chemical composition and qualitative characteristics of the *Longissimus* muscle of Nellore young bulls fed high-concentrate diet with different fiber sources.

	Fiber source		SEM	*P*-value
	WPCS	CSC		
DM (g/kg)	244.9	228.2	9.64	0.0974
Ash (g/kg)	8.7	8.3	0.58	0.5661
OM (g/kg)	236.2	220.6	9.49	0.1134
Protein (g/kg)	213.6	204.56	15.79	0.5730
Fat (g/kg)	22.5	24.1	6.24	0.8065

*WPCS* diet with whole plant corn silage as fiber source, *CSC* diet with cottonseed cake as fiber source, *DM* dry matter, *OM* organic matter, *SEM* standard error of the mean.

There was no interaction (*P* > 0.05) between fiber sources and maturation time (24 h and 7 days) for exudate losses, cooking losses, and the qualitative characteristics evaluated in the *Longissimus* muscle (Table 3). Shear force tended to a lower value (*P* = 0.0545) for longer meat aging time, about 7.27 N versus 8.49 N for 7 ds and 24 h of maturation, respectively. The WPCS diet tended to have greater positive yellow (b*) staining (*P* = 0.0637), 9.45 versus 8.57 for WPCS and CSC, respectively.

**Table 3:** Qualitative characteristics of the Longissimus muscle after 24 h and 7 days of maturation of Nellore young bulls fed Cottonseed Cake or Corn Silage in diets.

	WPCS		CSC		SEM	*P-*diet × maturation	*P*-diet	*P*-maturation
	24 h	7 days	24 h	7 days				
Exudation losses (g/kg)	49.27	59.84	46.09	51.64	6.0297	0.6800	0.3506	0.1883
Cooking losses (g/kg)	270.19	254.91	241.06	254.91	11.3258	0.1956	0.2159	0.9735
Shear force (N)	8.59	7.33	8.39	7.20	0.6179	0.9526	0.7876	0.0545
MFI	81.35	78.68	80.15	82.49	1.9841	0.2135	0.5154	0.9359
pH	5.85	5.88	5.94	5.92	0.0735	0.9890	0.3848	0.7203
L*	34.87	36.43	34.40	35.38	0.8786	0.7446	0.3911	0.1550
a*	14.81	14.42	14.53	14.30	0.3126	0.7846	0.5193	0.3311
b*	9.44	9.40	8.41	8.72	0.4465	0.7046	0.0637	0.7646

*WPCS* diet with whole plant corn silage as fiber source, *CSC* diet with cottonseed cake as fiber source, *MFI* myofibrillar fragmentation index, *SEM* standard error of the mean.

L* = luminosity; a* = red intensity; b* = yellow intensities; mean standard error.

The meat of young bull fed WPCS diet showed higher concentrations (*P* < 0.05) of C16:1, C18:1n9c, C18:3n3, and C22:2 (Table 4). Contrarily, the meat of animals fed CSC diet had higher concentrations (*P* < 0.05) of C15:0, C18:2n6c, and 20:3n3. The meat of animals fed the WPCS diet showed higher concentrations of monounsaturated fatty acids and ω9, while the CSC treatment showed higher concentrations of ω6 and ω6:ω3 ratio (Table 4).

**Table 4:** Fatty acid profile (mg/100 g of fat) of the *Longissimus* muscle of Nellore young bulls fed high-concentrate diet with different fiber sources.

	Fiber source		SEM	*P*-value
	WPCS	CSC		
C10:0	37.7	23.1	0.083	0.0929
C12:0	49.4	40.2	0.090	0.3243
C13:0	57.6	25.1	0.182	0.0907
C14:0	2598.0	2556.9	1.857	0.8271
C15:0	252.1	351.7	0.343	0.0086
C16:0	24,269.1	24,062.0	7.530	0.7858
C16:1	2841.2	2236.0	1.759	0.0025
C17:0	535.2	933.7	0.892	0.0002
C17:1	236.0	238.5	0.310	0.9287
C18:0	13,264.8	14,083.5	6.289	0.2070
C18:1n9c	38,896.8	28,746.6	14.719	0.0088
C18:1n9t	165.0	6,061.8	0.719	0.0001
C18:2n6c	5,556.7	9,207.7	11.501	0.0044
C18:2n6t	142.8	157.2	0.410	0.9287
C18:3n3	305.5	220.9	0.367	0.0309
C18:3n6	62.2	63.5	0.183	0.9476
C20:0	197.4	229.2	0.159	0.0586
C20:1	102.8	108.3	0.239	0.8141
C20:2	50.3	75.3a	0.105	0.0276
C20:3n3	1.0	72.9a	0.261	0.0109
C20:3n6	307.0	499.1	0.961	0.0590
C20:4n6	1,386.2	1,543.7	3.162	0.6236
C20:5n3	229.5	241.0	0.377	0.7639
C21:0	220.1	158.1	0.421	0.1576
C22:0	5.1	1.5	0.033	0.2845
C22:2	12.4	0.0	0.058	0.0463
C22:6n3	84.4	88.0	0.160	0.8212
C23:0	0.9	0.0	0.009	0.3502
C24:0	41.9	36.3	0.103	0.5914
Saturated fatty acids	41,529.0	42,502.0	11.269	0.3980
Monounsaturated fatty acids	42,242.0	37,392.0	15.623	0.0054
Polyunsaturated fatty acids	8,137.0	11,156.0	18.067	0.1090
ω3	535.0	535.0	0.822	0.9990
ω6	74.6	114.7	14.855	0.0133
ω9	39,062.0	34,808.0	14.719	0.0088
ω6:ω3	1,451.0	2,251.0	2.299	0.0023
Not identified	7,968.3	7,902.3	5.965	0.9130

*WPCS* diet with whole plant corn silage as fiber source, *CSC* diet with cottonseed cake as fiber source.

## Discussion

The cottonseed cake as a source of fiber promoted higher animal performance, despite having lower DM intake than the WPCS diet. Forages with low digestibility and high fiber content can reduce DM intake due to their low rate of degradation in the rumen and passage through the gastrointestinal tract, which can cause ruminal filling^29^. In addition to being a source of fiber, the cottonseed cake has satisfactory levels of CP (296.2 g/kg DM, Table 5), rumen undegraded protein, and lipids (81.8 g/kg DM, Table 5), being considered a source of physically effective fiber^29,30^, which may explain the better performance of cattle fed CSC diet.

**Table 5:** Chemical composition of the ingredients and of the experimental diets.

	DM	OM	CP	NDF	peNDF	EE	NFC
Ingredients	g/kg DM						
Whole plant corn silage	346.9	963.0	72.0	702.2	370.24	22.0	337.0
Cottonseed cake	935.5	956.1	296.2	670.5	466.14	81.8	97.0
Grounded corn	921.4	988.5	90.0	263.0	-	31.8	723.0
Soybean Bran	941.5	928.4	480.0	164.1	-	3.9	276.0
Extruded urea	961.0	995.7	2029.7	32.5	-	31.6	218.0
Mineral	990.0	–	–	–	–	–	–
Diets	g/kg DM						
Corn silage (WPCS)	738.0	953.9	185.8	368.8	182.8	23.4	507.0
Cottonseed cake (CSC)	911.5	959.9	183.5	374.7	230.9	45.1	500.0

*WPCS* diet with whole plant corn silage as fiber source, *CSC* diet with cottonseed cake as fiber source, *DM* dry matter, *OM* organic matter, *CP* crude protein (N total × 6.25), *NDF* neutral detergent fiber, *peNDF* physically effective neutral detergent fiber, *EE* ethereal extract, *NFC* non-fiber carbohydrate.

Although bulls fed CSC exhibited higher CY, the same difference was not observed when assessing the GY as depicted in Table 3. This discrepancy can be attributed to the influence of diet type, particularly the quantity and nature of fiber, on the size and weight of ruminants gastrointestinal tracts, thereby affecting CY values^31^. Factors contributing to this phenomenon include the higher concentrations of DM, NDF, and peNDF in the CSC diet. Moreover, a related study revealed that despite CSC having higher peNDF (particles ≥ 8.0 mm), bulls fed CSC exhibited approximately 70% more rumination time than those fed CSC^21^^.^ The CSC diet likely entails a shorter retention time in the rumen and potentially a higher passage rate, leading to reduced growth of gastrointestinal tract organs.

Carcass finish, the internal and external depth, the physiological maturity, and the conformation observed can be considered adequate according to Gomes et al*.*^32^. The animals fed WPCS presented REA 11.4% higher than those fed CSC diet. Barducci et al*.*^30^ not observed differences in the REA of cattle finished with a naturally protected fat source considering cottonseed cake, commercial protected fat (Megalac-E^®^), and without a protected fat source.

The fiber sources had no effect on the meat's degree of marbling. Miyaki et al*.*^33^ found about half of the marbling results (4.7) of the present study when evaluating diets with oilseeds for Nellore young bulls finished in feedlot. These values are in line with expectations, as the presence of intramuscular fat in Zebu cattle is very small, in addition to its deposition occurring later than subcutaneous fat^5^.

The luminosity (L*), positive red (a*), and positive yellow (b*) values of muscle and subcutaneous fat showed no significant differences between diets. Animals finished on pasture or with a higher proportion of forage in their diet typically exhibit more intense coloring of both meat and fat, attributed to the increased consumption of nutrient-rich forage containing higher concentrations of pigments and carotenoids^7^. Hence, the absence of any influence of the fiber source on meat color serves as further evidence that CSC can effectively substitute traditional roughage in the feedlot steers diet.

Thawing (exudate) and cooking losses were not altered by the fiber source, probably because it also had no effect on subcutaneous fat thickness (SFT). Likewise, Leketa et al*.*^17^ also did not observe differences in exudate and cooking losses in the meat of castrated Saanen young goats fed diets with different protein sources, diets with a mixture of oilseeds (soybean, sunflower cake, and cottonseed cake) versus the diet with Leucaena hay and oil seed cake protein (sunflower cake and cottonseed cake).

The maturation by 7 days promoted a reduction of 14% in the shear force. Probably, maturation promoted a reduction in shear force by breaking down the muscle fibers and weakening the myofibrillar connections. This reduction is due to an increase in the activity of proteolytic enzymes in the muscle, mainly calpain^34,35^. Eiras et al*.*^36^ observed a decrease in shear force with longer maturation times (24 h, 7 days, and 14 days).

Both fiber sources had no effect on muscle composition. Assis et al*.*^19^ also found no changes in moisture, protein, or total lipid in the meat of crossbred Boer goats fed with replacement levels of soybean meal per cottonseed cake (0, 33%, 66%, and 100%). However, Eiras et al*.*^36^ observed a reduction in lipid content with greater inclusion (33%) and greater moisture with the inclusion of 27% cotton hulls in the diet of crossbred steers (½ Simmental × ½ Nellore).

The highest concentration of linoleic acid (C18:2n6) was found in the meat of young bulls fed CSC diet, and the highest concentration of oleic acid (C18:1n9) in animals fed WPCS diet. These findings can be attributed to the higher abundance of these fatty acids in the respective fiber sources. As noted by several authors, dietary manipulation stands as an effective method for altering the fatty acid profile. For instance, Miayaki et al*.*^33^ observed elevated levels of C18:1n9 in the meat of animals not supplemented with oilseeds. Similarly, Rodrigues et al*.*^23^ reported increased concentrations of C18:2n6c in the *Longissimus* muscle of sheep fed cottonseed compared to those fed soybeans or a diet devoid of oilseeds. Machado Neto et al*.*^37^ noted a higher concentration of C18:2n6 in cottonseed, while corn silage and soybean seed led to elevated levels of C18:1n9, resulting in increased C18:2n6 and decreased C18:1n9 concentrations in beef meat from animals fed cottonseed.

In a review of several studies evaluating the quality of meat, Van Elswyk and McNeill^38^ observed that the ω6-linoleic acid (18:2n6), is the main fatty acid detected in the meat of steers both finished in pasture and in confinement. However, the diet can alter the ω3 concentrations in meat, as cited by Elswyk and McNeill^36^. The ω3-linolenic acid (18:3n3) ranges from 16 to 26 mg/g of fat in various lean cuts for grass-finished cattle while the fat for grain-finished cattle shows 4–13 mg/g. The same authors also reported that meat from grass-finished steers contains 30–70% less monounsaturated fatty acids than grain-finished. Thus, the total amount of polyunsaturated fatty acids in meat from grass-fed/forage-fed cattle can be up to 75 mg lower than grain-fed cattle. Our results showed a higher concentration of Linoleic acid, of polyunsaturated fatty acids, ω6, and in the ω6: ω3 ratio in the meat of animals fed cottonseed cake as a fiber source. Contrarily, Cama-Moncunill et al.^39^ observed that the meat of cattle fed forage had higher concentrations of polyunsaturated fatty acids and ω3, while those fed concentrates had higher ω6 content and higher ω6:ω3 ratio in meat. Our findings are directly linked to meat quality for the benefit of human health. Therefore, we can confirm the hypothesis that young bulls fed cottonseed cake present meat rich in polyunsaturated fatty acids. Furthermore, cottonseed cake as a fiber source can improve the quality of the final product for human consumption. The intake of polyunsaturated acids provides a significant decrease in cholesterol levels and has positive actions to increase tocopherol levels, in addition to improving insulin sensitivity^40,41^.

## Conclusion

Incorporating cottonseed cake as a fiber source into the diet, even without forage, leads to higher concentrations of polyunsaturated fatty acids and ω6 fatty acids in the meat of feedlot-finished young bulls. This suggests that cottonseed cake can be effectively utilized in diets lacking forage to increase quality of carcass and meat in confined cattle.

## Material and methods

The experiment was carried out between October 2020 to January 2021, totaling 112 days of feedlot in the Beef Cattle Confinement Sector of the School-farm of the Federal University of Mato Grosso do Sul (UFMS), located in the municipality of Terenos-MS, Brazil, which have an Aw—tropical savanna climate with a dry season ranging from four to five months – according to the *Köppen–Geiger* climate classification^42^.

This research was conducted in strict accordance with the recommendations of the National Council for the Control of Animal Experiments Guide. The experimental research protocol was approved by the Ethics Committee on the Use of Animals of the Universidade Federal do Mato Grosso do Sul (Protocol 1.181/2021). Study is reported in accordance with The ARRIVE (Animal Research: Reporting of in vivo Experiments) guideline^43^.

A completely randomized design was employed with two treatments (fiber sources) and 12 replications (young bulls). Twenty-four Nellore young bulls (*Bos taurus indicus* L.) with an average initial body weight (IBW) of 377.8 ± 43.5 kg and approximately 24 months old, from the School-farm herd of UFMS, were individually housed in pens and fed separately. The treatments consisted of two experimental diets (Table 5), with different fiber sources. The whole plant corn silage (WPCS; *Zea mays* L.) and, cottonseed cake (CSC; *Gossypium* spp. L.) as fiber sources were added on a 300 g/kg dry matter basis, in both diets.

The diets (Table 5) were carefully balanced to ensure uniform levels of CP (180 g/kg DM) and NDF (370 g/kg DM), targeting an average daily gain of 1.5 kg and a slaughter weight of 530 kg. Each diet consisted of 30% forage, primarily in the form of corn silage, while the forage-free diet incorporated 30% cottonseed cake as a fiber source, all calculated on a DM basis. The animals were fed twice a day, in the morning (09:00) and in the afternoon (14:00), for intake of approximately 2.4% of body weight, and approximately 10% leftovers.

Ingredients and total diets were sampled to determine DM, MM, OM, CP, NDF, and ethereal extract (EE), according to Detmann et al*.*^44^. To estimate the non-fiber carbohydrate (NFC) content, the equation proposed by Sniffen et al*.*^45^:

$$NFC \left(\%\right)= 100- (NDF + CP + EE + MM).$$

The peNDF of both fiber sources (WPCS and CSC) were determined by the laboratory method of the Penn State Particle Separator proposed by Lammers et al*.*^8^ (Table 5). In this work, the NDF multiplied by the DM of the samples retained in trays with 19 and 8 mm sieves was considered. The peNDF of each fiber source present in the roughage concentration was considered to determine the peNDF of diets (Table 6).

**Table 6:** Fatty acid profile (mg/100 g of lipid) of feeds and hyperconcentrated diets with different sources of fiber for cattle in confinement.

	Ground corn	Soybean bran	Corn silage	Cottonseed cake
C14:0	1.0	–	–	–
C16:0	16,280.0	20,640.0	15,320.0	12,580.0
C17:0	–	–	–	1830.0
C17:1	–	–	4.0	–
C18:0	2720.0	3820.0	860.0	–
C18:1n6	–	51,230.0	–	–
C18:1n9	23,740.0	16,580.0	7.750,0	8580.0
C18:2n6	45,910.0	–	17,640.0	30,410.0
C18:3n3	2390.0	4820.2	17,810.0	2820.0
C18:3n6	–	–	6.23,0	–
C20:3n3	–	–	1.080,0	–
Unidentified	8959.0	2910.0	33,280.0	43,790,0

At the end of the experimental period of confinement (112 days), the animals were fasted for 24 h and sent for slaughter in a commercial slaughterhouse. The carcasses were individually identified, longitudinally sectioned, weighed, and cooled at 2 °C for 24 h. The CY as a function of the HCW and the fasting FBW of the animals as described by Gomes et al*.*^32^:

$$CY = (HCW/FBW \times 100)$$

The GY was estimated according to Moretti^46^, being calculated by dividing the carcass gain by the body weight gain, in %:

$$GY = [(final\, HCW- initial\, HCW)/(FBW- IBW)] \times 100$$

The value of the initial hot carcass weight was estimated at 50% of the initial body weight (IBW ÷ 50).

After 24 h of cooling, the left half of the carcass was evaluated for finishing measures, length and depth, physiological maturity, conformation, REA, fat distribution, the SFT, carcass pH, meat color and fat, went determined as described by Gomes et al*.*^32^.

For the carcass finishing score, the observation scale of the distribution and amount of subcutaneous fat at the height of the 6th, 9th, and 12th ribs were as follows: Absent (1.0 ± 0.3), Scarce (2.0 ± 0.3), Median (3.0 ± 0.3), Uniform (4.0 ± 0.3), and Excessive (5.0 ± 0.3).

The measurements of internal depth and external depth of the half carcass were carried out using the distances between the cranial edges of the ischiopubic symphysis and the first rib; between the lower edge of the medullary canal at the height between the 5th and 6th thoracic vertebrae to the edges of the sternum taken internally or externally to the carcass, respectively.

The determination of the physiological maturity score was conducted by observing the ossification of the cartilage present in the thoracic, lumbar, and sacral vertebrae, as well as the color and shape of the ribs and the color of the meat, at the level of their respective vertebrae. A scoring scale divided into classes A to E (− 0 +) was employed as follows: A represents cattle aged between 9 and 30 months, B: 30–42 months, C: 42–72 months, D: 72–96 months, and E: above 96 months.

Carcass conformation was evaluated in increasing order of muscularity with variation (+ 0 −) that were converted into numerical values, where: Convex with values from 15 to 13; Sub-convex from 12 to 10; Rectilinear from 9 to 7; Sub-rectilinear from 6 to 4, and Concave from 3 to 1.

After finishing and conformation evaluations, the Longissimus muscle was sectioned between the 12th and 13th ribs, exposing the rib eye area (REA). Subsequently, the outline of the REA was drawn on tracing paper, which was read in a leaf area meter LI-3100C (Li-Cor Inc., LI, USA). The degree of marbling was evaluated from specific photographic scores, with variation (− 0 +), in the cross-section of the *Longissimus* muscle (between the 12th and 13th ribs). The photographic scales were placed close to the cut for evaluation, and then, the value of the scale that most closely matches the observed marbling.

Carcass pH was measured using a portable pH meter equipped with specific carcass penetration probes, model HI99163 (Hanna Instruments, Woonsocket, USA). The instrumental color aspects of meat and subcutaneous fat on the *Longissimus* muscle 24 h *post-morten* were evaluated using a MiniScan XE Plus colorimeter (HunterLab, Reston, USA) programmed to measure the parameters of the CIELab system: luminosity (L*) which is a measure of reflected light (100 = white, 0 = black); red intensities (a*) which measure positive-red and negative green; and yellow (b*) measures positive-yellow and negative-blue. The mean resulting from the measurements taken at three sample points was recorded. The SFT was measured using a digital caliper, from the edge adjacent to the animal's spine, in which: < 1.0 mm absence fat, 1–3 mm scarce fat, 3–6 mm median fat, 6–10 mm uniform fat and > 1.0 mm excessive fat.

For the fat distribution in hindquarters, three classes were considered, subdivided into subclasses (− 0 +), in which: 1 represents the rear with poor fat distribution; 2 represents an intermediate distribution, and 3 is the best one, where all the cuts of the hindquarters are covered by fat.

Meat quality analyses were performed in the Applied Nutrition Laboratory and the Meat Quality Laboratory at Federal University of Mato Grosso do Sul. The analysis of the fatty acid profile of food and meat was carried out at the Multipurpose Laboratory and the Laboratory of Analysis of Gases and Oils of the Faculty of Chemistry, at Federal University of Mato Grosso do Sul, located in the municipality of Campo Grande-MS, Brazil.

Protein, moisture, and muscle ash contents were determined according to AOAC^47^. Protein was determined by the Kjeldahl method with a step of digestion in sulfuric acid solution and catalytic mixing, distillation, and titration in hydrochloric acid solution. The DM was determined by drying for 12 h in an oven at 105ºC and later the samples were incinerated in muffle oven at 600 °C for 4 h. The intramuscular fat (EE) content was quantified according to AOAC^47^, using an automatic extraction system (ANKOM XT15 Extractor, ANKOM Technology, Macedon, USA), and consisting of two steps: first, sample preparation by drying in an oven for 15 h at 105 °C and second, extraction with petroleum ether at high temperature and pressure. Meat OM was determined by subtracting the MM content.

Two 2.5 cm thick muscle samples were identified, packaged, and frozen at − 20 °C after 24 h of maturation. Two other samples were submitted to maturation for seven days (7 d) at 4 °C and subsequently stored at − 20 °C for determination of pH, color (L*, a*, and b*), cooking losses, shear force, and myofibrillar fragmentation index.

Exudation and cooking losses were determined according to AMSA^48^. The samples were thawed in a cold chamber at 2.0 °C (± 2.0) for 24 h before the analysis procedures. Before removing the steaks from the packaging, from the 24 h and 7 days of aging samples, the exudate was weighed. Subsequently, the pH in the central region of the steak and the colors (L*, a*, and b*) of the aged steaks (24 hs and 7 ds) were measured^33^. Calibration of the pH meter was performed using two buffers with pH 4.01 and 7.0. Temperature compensation in the range of − 5–105 °C on the measuring device is automatic.

Then, the steaks were roasted in an electric oven at 163 °C (Layr, model Crystal, with upper and lower heating elements, São Paulo, Brazil). The internal temperature was monitored with thermocouples (Taylor, model 1478-21, Ohio, USA), inserted in the geometric center of the samples, and removed from the oven when they reached 71 °C. The cooking loss was determined as the difference between the weight of the sample before cooking and the weight after cooling to room temperature.

Before the analysis of the shear force, the samples were cooled at room temperature, until the temperature of the steaks reached about 23 °C, and then they were sealed in plastic film and stored in a refrigerator (2–5 °C) for 24 h. Subsequently, six subsamples (1.27 cm) were taken, in the direction of the muscle fiber, from each sample with a metal anchor bolt adapted to an electric bench drill (©Schulz Pratika). The shear force was determined in a texture analyzer (CT3 Warner Bratzler, Brookfield Engineering, USA) and the shear force of the samples were calculated by the average obtained from six subsamples.

The analysis of the myofibrillar fragmentation index (MFI) of the samples of aged meat (24 h and 7 days) was performed according to the procedures of Culler et al*.*^49^. Fat and excess connective tissue were removed from the meat and the protein concentration of the myofibril solution was determined by the biuret method. Dilution (0.5 mg/mL) in a final volume of 8 mL was used for MFI determination. Absorbance at 540 nm was measured in an M SP-22 digital spectrophotometer (Agilent Technologies Inc., Palo Alto, CA, USA). The MFI was calculated with the absorbance found at 540 nm multiplied by the constant 200 and the result was expressed in units.

The extraction of lipids from the ingredients (corn, soybean meal, CSC, and WPCS) from the total rations (Table 6) and from the meat was by the method of Bligh and Dyer^50^, using a methanol-chloroform mixture at 35 °C in 12 rpm for 15 min. Extraction and evaluation of total lipids were performed with hexane and isopropanol in a 3:2 ratio. In the transesterification of fatty acids using the methanolic solution of sodium methoxide.

The fatty acid profile of meat was performed by high-performance gas chromatography. A gas chromatograph equipped with an SP-2560 capillary column with a length of 100 m and a diameter of 0.25 mm coupled to a flame ionization detector was used. Helium gas was used in the mobile phase.

Data were submitted to analysis of variance using the PROC GLM procedure of the SAS statistical package (SAS University Edition, SAS Institute Inc. Cary, CA, USA). To compare the means of the variables and the effect of the diets, the IBW was considered as a covariate. The F test was used at 0.05 of significance. The statistical model used was the following: $Yij = \mu + ti + \beta (X - Xij) + eij$, where: *Y*_*ij*_ is the value observed in treatment *i* and in repetition *j*; *μ* is the general mean; *t*_*i*_ is the treatment effect (*i* is WPCS or CSC); *β (X – X*_*ij*_*)* is the effect of the covariate (initial body weight); and *e*_*ij*_ is the random error associated with each observation.

## Data Availability

All data generated or analyzed during this study are included in this published article.
